# Integrated Clinical Workflow for Preoperative Planning and Resection of Giant Iliofemoral Heterotopic Ossification Using Three-Dimensional Technologies

**DOI:** 10.3390/jcm15051893

**Published:** 2026-03-02

**Authors:** Arpad Solyom, Janos Szekely, Liviu Moldovan, Flaviu Moldovan

**Affiliations:** 1 Orthopedics—Traumatology Department, Faculty of Medicine, George Emil Palade University of Medicine, Pharmacy, Science, and Technology of Targu Mures, 540142 Targu Mures, Romania; arpad.solyom@umfst.ro; 2 IOSUD Doctoral School, George Emil Palade University of Medicine, Pharmacy, Science, and Technology of Targu Mures, 540142 Targu Mures, Romania; egyjanek@yahoo.com; 3 Faculty of Engineering and Information Technology, George Emil Palade University of Medicine, Pharmacy, Science, and Technology of Targu Mures, 540142 Targu Mures, Romania; liviu.moldovan@umfst.ro

**Keywords:** heterotopic ossification, spinal cord injury, 3D printing, surgical planning, neurovascular preservation, iliofemoral ankylosis

## Abstract

**Background/Objectives**: Neurogenic heterotopic ossification (HO) is an abnormal formation of lamellar bone in soft tissues, frequently developing near major joints in patients with spinal cord injury. While imaging provides valuable diagnostic insights, large and anatomically complex HO often requires advanced preoperative planning to minimize surgical risks. This study presents the development and clinical application of a structured six-stage workflow integrating three-dimensional (3D) technologies for the preoperative planning and surgical resection of giant iliofemoral HO. **Materials and Methods**: A workflow was developed comprising: (1) 3D imaging acquisition, (2) creation of a virtual model, (3) production of a life-size physical model, (4) preoperative simulation, (5) surgical resection, and (6) postoperative imaging validation. The workflow was applied to a 50-year-old male with paraplegia after a T12 fracture who developed a 26 cm iliofemoral bony bridge, confirmed by computed tomography and 3D reconstruction. **Results**: The physical model provided a precise anatomical reference, enabling detailed surgical rehearsal and safe planning of neurovascular dissection. Resection was performed using combined orthopedic and vascular techniques. The hip joint was preserved, and postoperative rehabilitation achieved improved range of motion and patient handling without major complications. **Conclusions**: This structured 3D-assisted workflow enhanced anatomical understanding and surgical precision in this complex case. The framework is applicable to other extensive ossifications with intricate anatomical relationships and warrants further evaluation in larger series.

## 1. Introduction

Heterotopic ossification (HO) is defined as the pathological formation of mature, lamellar bone in soft tissues at sites where bone does not normally exist [1]. It represents a complex and incompletely understood biological process involving the inappropriate differentiation of mesenchymal stem cells into osteoblasts, often triggered by local or systemic factors [2]. HO is encountered in a range of clinical contexts, including after trauma, surgery, neurological injury, or as part of rare congenital syndromes [3]. Neurogenic HO is a particularly challenging subtype that occurs after injuries to the central nervous system, such as traumatic brain injury or spinal cord injury (SCI) [4]. Reported incidence varies between 10 and 20% in SCI patients, with the hip joint being the most frequently affected site—accounting for up to 90% of cases [5]. Factors that increase the risk of HO in SCI include high-energy trauma, thoracic injury, severe spasticity, pneumonia, urinary tract infection, prolonged immobilization, and the presence of a tracheostomy [6].

The pathogenesis of HO is multifactorial and incompletely elucidated. Contributing mechanisms include tissue hypoxia, microtrauma from patient handling, vascular stasis, and inflammatory cytokine release [7]. These create a permissive environment for osteogenic precursor cells to differentiate and form ectopic bone [8]. The ossification process evolves through three distinct phases: early, when soft tissue calcifications appear, often mistaken for infection or tumor; intermediate, when lamellar bone begins to form but is not yet fully mineralized; and mature, when fully formed cortical and cancellous bone is present, potentially bridging adjacent skeletal structures [9]. Clinically, HO can cause progressive pain, swelling, reduced range of motion, and—in advanced cases—complete ankylosis of the involved joint [10]. For SCI patients, the consequences extend beyond mobility loss to include impaired hygiene, increased risk of pressure ulcers, and difficulty with transfers or wheelchair positioning. These functional limitations directly compromise rehabilitation potential [11].

Early diagnosis is difficult because initial clinical signs overlap with infection or deep vein thrombosis, and imaging findings may be non-specific. While Magnetic Resonance Imaging (MRI) can demonstrate early soft tissue changes, it lacks specificity for immature HO. Computed Tomography (CT) is the gold standard for defining mature ossification and assessing its anatomical relationships, particularly to neurovascular structures [12]. Serum alkaline phosphatase (AP) levels may correlate with HO activity but are not definitive for maturity assessment [13]. Medical prophylaxis, such as nonsteroidal anti-inflammatory drugs (NSAIDs), selective Cyclooxygenase-2 (COX-2) inhibitors, or low-dose radiotherapy, can reduce the incidence of HO when applied early [14]. However, mature HO with significant functional impairment requires surgical resection [15]. The timing of surgery is critical: intervention before maturation risks recurrence, whereas excessive delay prolongs disability. Intraoperatively, the surgeon needs standard two-dimensional (2D) views, as well as those of the acetabulum and proximal femur [16]. Because of the anatomy and overlapping structures, evaluation of 2D views may yield limited information. For this reason, intraoperative three-dimensional (3D) imaging has become common in the last decade [17]. However, the orthopedic implants used may affect radiographic evaluation. This disadvantage is limited by intraoperative CT imaging which provides improved image quality and allows for a considerably expanded field of view [18].

Recent developments in 3D imaging and printing have revolutionized orthopedic preoperative planning. By generating patient-specific virtual and physical models, surgeons can visualize complex pathologies, simulate surgical steps, and anticipate technical difficulties [19]. These models improve intraoperative orientation, reduce operative time, and facilitate multidisciplinary collaboration [20]. While widely adopted in fracture surgery, tumor resection, and arthroplasty, applications to HO—particularly large, neurovascularly complex cases—remain limited in the literature [21].

Three-dimensional (3D) printing encompasses a variety of manufacturing methods that transform digital data into tangible objects [22]. The development of polymer-based nanocomposites has further enhanced the capabilities of this technology [23], enabling broader applications in healthcare. In medicine, it provides new opportunities for creativity and extends the value of diagnostic imaging by facilitating anatomical model reconstruction [24]. Medical imaging techniques are therefore essential in producing accurate 3D-printed models [25]. Within orthopedics, the use of this technology is rapidly growing, particularly for preoperative planning, the design of orthoses, the production of patient-specific implants and surgical guides, and its contributions to precision and personalized treatment approaches [26].

Solyom et al. [27] have validated a six-stage 3D-assisted clinical workflow previously applied to complex acetabular fractures that can be further developed and validated for the resection of giant iliofemoral heterotopic ossification. Selemen et al. [28] have developed a new surgical technique composed of a navigation system and a 3D imaging tool which is added to the classic hip osteoma resection. Toga et al. [29] used a curved chisel for the resection of heterotopic ossification whose profile can be defined prior to the intervention by using a 3D-printed model.

With the support of these findings revealed from the scientific literature regarding the resection of giant iliofemoral heterotopic ossification, in this study we formulated the following research question: How can three-dimensional technologies be integrated in the current practice of preoperative planning and what is the clinical workflow for the resection of giant iliofemoral heterotopic ossification?

The objective of our research is to develop and validate a medical workflow integrated with 3D-printing technologies for preoperative planning and surgical intervention in giant iliofemoral heterotopic ossification in clinical practice.

In this proof-of-concept study, we developed a structured six-stage workflow and validated its clinical applicability through its detailed application to a single complex case, establishing feasibility for future larger-scale implementation.

## 2. Materials and Methods

### 2.1. Overview of the Clinical Workflow

This study presents the development and proof-of-concept validation of a structured clinical workflow using a single patient case to demonstrate feasibility and reproducibility. The latest advances in three-dimensional (3D) technologies integrate imaging, digital modeling, numerical simulation, and rapid prototyping. These systems employ specialized software capable of analyzing tissues, extracting relevant data, and thereby enhancing the efficiency of medical evaluations [30]. Within orthopedics, this suite of 3D tools supports more precise diagnosis; improves preoperative planning; and facilitates the development of tailored surgical instruments, patient-specific guides, and custom implants [31].

**Figure 1:**
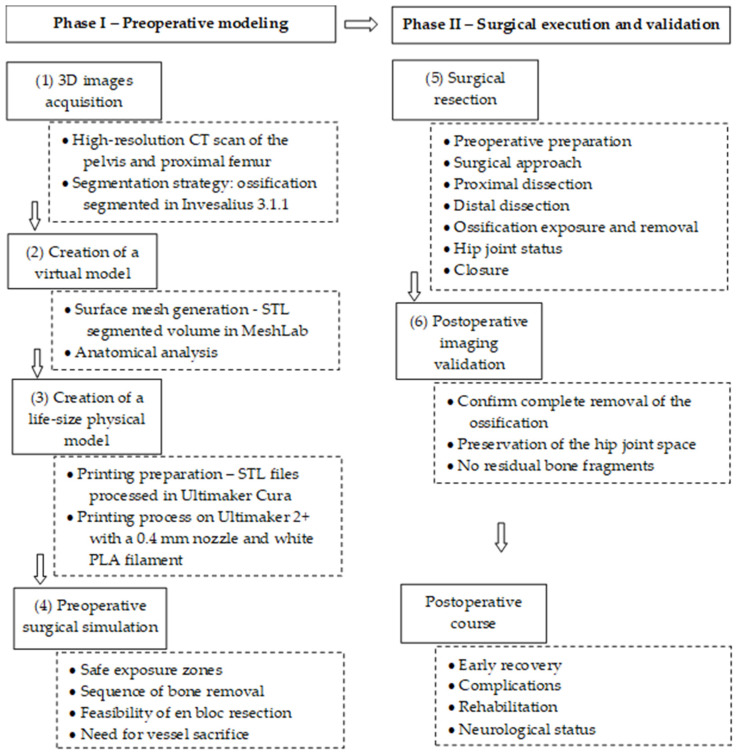
Integrated clinical workflow for preoperative planning and resection of giant iliofemoral heterotopic ossification using three-dimensional technologies.

In cases of giant iliofemoral heterotopic ossification (HO), the critical factors for surgery are the maturity of the bone, which is best assessed with CT scans, and the presence of significant functional limitation due to the HO. Surgery is considered when the HO is mature and significantly restricts range of motion, with the goal of restoring function. The original injury must be stable, and the patient’s overall medical condition should be optimized to reduce the risk of complications. Surgery applications are supported by the structured integration of new 3D technologies [32]. For this purpose, by integrating 3D printing technologies, we developed a clinical workflow for the resection of giant iliofemoral heterotopic ossification (Figure 1). It contains six stages grouped into two distinct phases: Phase I—Preoperative modeling; Phase II—Surgical execution and validation. In the first period, modeling is executed first virtually, and then the physical model is created, as follows:

Phase I—Preoperative modeling

- Virtual modeling (VM)
  (1) 3D imaging acquisition.
  (2) Creation of the virtual HO model.
- Physical modeling (PM)
  (3) Creation of the physical HO model.
  (4) Preoperative surgical simulation.

Phase II—Surgical execution and validation

  (5) Surgical resection.
  (6) Postoperative imaging validation.

### 2.2. Stage 1—3D Imaging Acquisition

A high-resolution CT scan of the pelvis and proximal femur was obtained with angiographic sequences to delineate the course of the iliac and femoral vessels relative to the ossification. Slice thickness was set at 0.625 mm to maximize spatial resolution, and multiplanar reconstructions were reviewed. DICOM (Digital Imaging and Communications in Medicine) data were exported for segmentation.

The segmentation strategy employed the Invesalius (version 3.1.1, CTI, Brasil) software [32] when bone tissue was identified by thresholding between 700 and 2500 HU (Hounsfield Units) for cortical bone and ~700 HU for cancellous bone. The ossification was segmented as a single continuous volume extending from the inner surface of the iliac bone, beneath the inguinal ligament, to the proximal femoral shaft.

### 2.3. Stage 2—Creation of the Virtual Model

Creation of the virtual model continues with digital image processing. For surface mesh generation the segmented volume was converted into an STL (Standard Tessellation Language or Stereolithography) file and imported into MeshLab (version 2023.12, ISTI—CNR, Pisa, Italy) [33] and FreeCAD (version 0.21.2, LGPL, FSF, Boston, MA, USA) [34] for artifact cleaning. Small, disconnected elements were removed, holes were filled, and mesh smoothing was applied to improve model integrity while preserving key surface features.

Next, we performed an anatomical analysis. The virtual model allowed for rotation, sectioning, and measurement of the ossification. Maximum length was 26 cm, with a width of 8.56 cm at its broadest point. Medial spike-like projections were noted, oriented toward the pelvic cavity. The model clearly demonstrated displacement of the femoral neurovascular bundle anteromedially.

### 2.4. Stage 3—Creation of the Physical Model

Three-dimensional printing of the giant iliofemoral heterotopic ossification allows for obtaining a physical model with the support of which the patient’s condition can be explored in detail. The coded model in STL format is used for 3D printing of the life-size physical model, as these files describe, in mathematical form, the geometry of the three-dimensional bone surface.

In the printing preparation phase the STL file was processed in Ultimaker Cura (version 4.10., Ultimaker B.V., Netherlands) [35] with the following parameters: layer thickness: 0.2 mm; wall thickness: 1.2 mm; infill density: 10%; printing speed: 50 mm/s; build plate adhesion: brim; and support: everywhere, to preserve overhanging structures. In the printing process an Ultimaker 2+ [36] with a 0.4 mm nozzle and white Poly Lactic Acid (PLA) filament (2.85 mm diameter) was used. The physical model reproduced fine surface irregularities and allowed for direct measurement and tactile examination.

### 2.5. Stage 4—Preoperative Simulation

The printed model was used in multidisciplinary sessions involving orthopedic and vascular surgeons [37]. The goals of the simulation were: determine safe exposure zones for vessel identification and looping; plan the sequence of bone removal (proximal to distal vs. distal to proximal); evaluate the feasibility of en bloc resection (determined to be impractical); and anticipate the need for vessel sacrifice (deep femoral artery). The simulation also allowed for the rehearsal of instrument placement, clamp positioning, and bone chiseling angles, reducing uncertainty during live surgery.

### 2.6. Workflow Validation

To demonstrate the clinical application of the proposed six-stage workflow, we present the case of a 50-year-old male patient admitted to the Orthopedic Department of the Targu Mures County Emergency Clinic Hospital [38]. The patient sustained a fall from 3 m, resulting in multiple injuries, including a T11–T12 fracture with traumatic anterolisthesis and consequent paraplegia, as well as cranial and thoracic trauma. Following emergency neurosurgical intervention and posterior spinal stabilization, the patient developed progressive right hip ankylosis due to heterotopic ossification. CT imaging revealed a 26 cm iliofemoral bony bridge. The structured workflow described in Section 2.1, Section 2.2, Section 2.3, Section 2.4 and Section 2.5 was applied to this patient to guide preoperative planning and surgical resection.

## 3. Results

**Figure 2:**
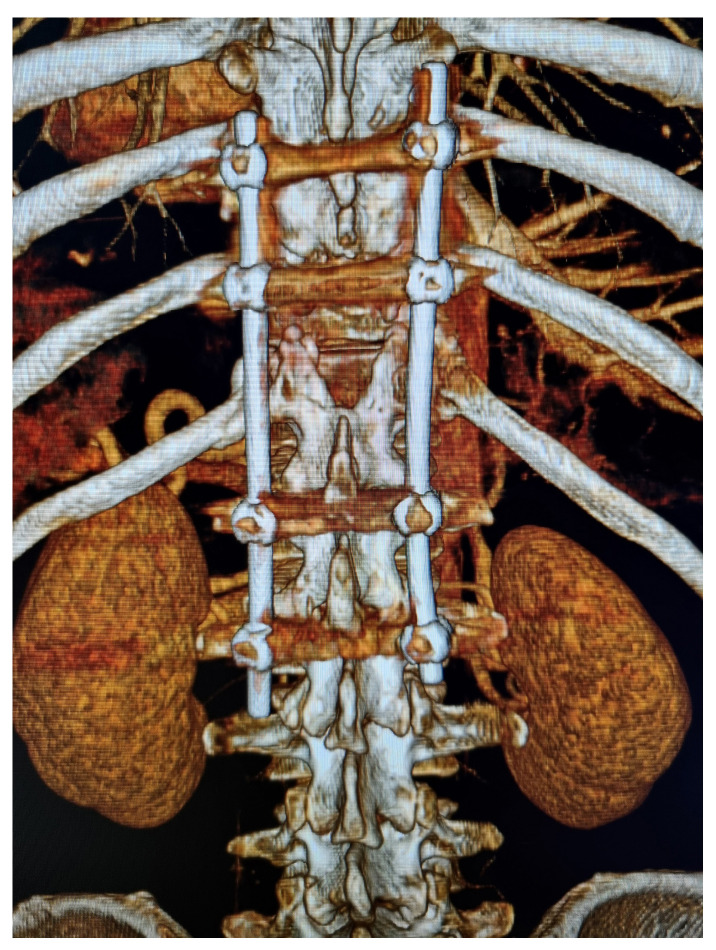
Posterior transpedicular stabilization of the thoraco-lumbar spine: CT reconstruction.

Because of the life-threatening, massive epidural hematoma, the patient underwent an emergency craniectomy with evacuation of the epidural hematoma followed by a posterior stabilization of the thoraco-lumbar spine with transpedicular instrumentation between T10 and L2 with decompressive laminectomy at the T12 level (Figure 2).

**Figure 3:**
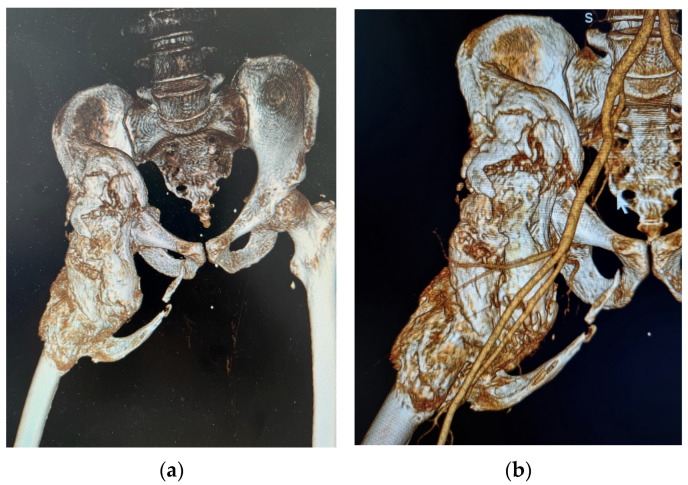
3D reconstruction of preoperative CT imaging in RadiAnt DICOM for the patient included in the study: (**a**) native window; (**b**) arterial window.

After one month of rehabilitation, the therapist complained of passive range of motion (ROM) limitation in the right hip. The radiography showed incipient periarticular HO, with an otherwise intact hip joint. At this time, the serum Alkaline Phosphatase level was normal (63 U/L). After that the passive ROM of the right hip worsened. Repeated radiographies of the joint showed development of the HO. Then the hip was fixed in external rotation, 30° of flexion and abduction. Paraplegia was present with a sensory loss about 5 cm below the umbilicus. The CT scan revealed a huge 26 cm long bony bridge with the widest point of 8.56 cm between the internal surface of the iliac bone passing below the inguinal ligament and lateral to the neurovascular bundle to the proximal third of the femur, consisting of mature bone with uneven shapes and surfaces and with medial spike-like extensions. The femoral neurovascular bundle is left outside but pushed antero-medially according to the Angio-CT performed (Figure 3).

**Figure 4:**
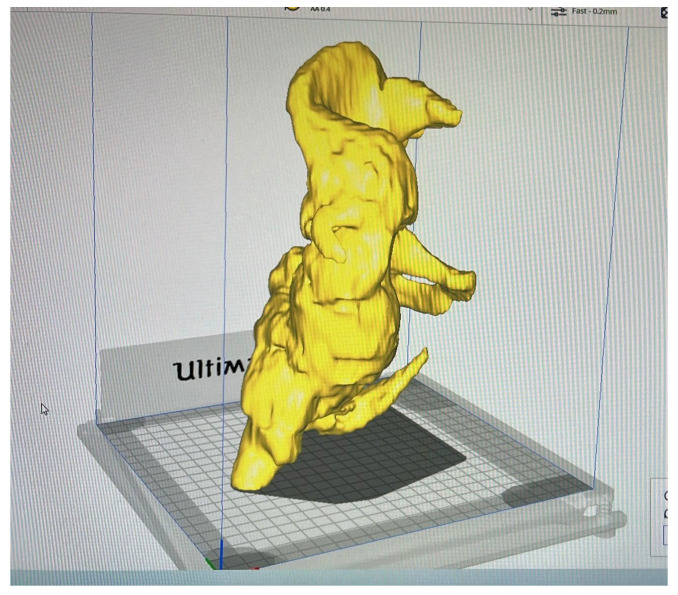
The virtual model of the giant iliofemoral heterotopic ossification prepared for printing in Ultimaker Cura software.

Although HO is a musculoskeletal disorder, because of its huge volume and complex anatomic relationships, we decided to manage it interdisciplinarily. To further assess the precise form and dimensions based on CT scans, we created a 3D virtual model of the HO. Figure 4 shows the results of the STL (Standard Triangle Language) segmentation process, which is a virtual model of the giant iliofemoral heterotopic ossification. As STL files mathematically describe the geometry of a three-dimensional bone surface, the model is prepared for 3D printing.

**Figure 5:**
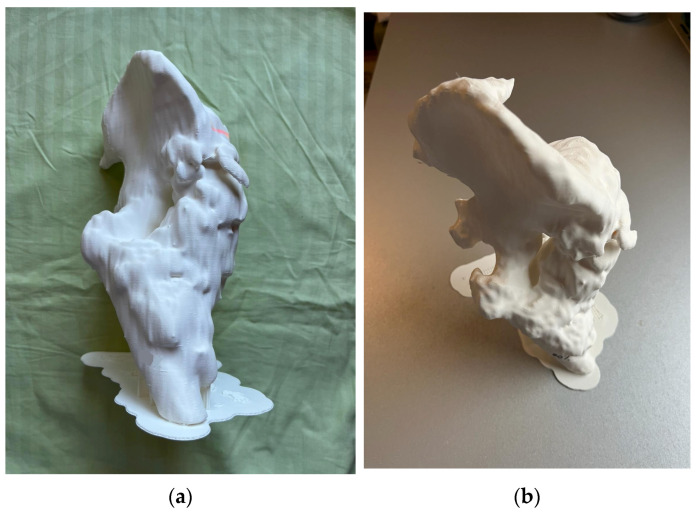
3D-printed life-size HO model showing complex irregular surfaces and medial spike-like projections: (**a**) anterior view; (**b**) cranial view.

3D printing was achieved through an FDM (Fused Deposition Modeling) additive manufacturing process, which creates three-dimensional objects by thermoplastic extrusion of filaments (Figure 5). The print time was 19 h and 23 min.

### 3.1. Stage 5—Surgical Resection

At this stage, orthopedic surgery relies on the outcomes of the preoperative simulation. Initially, the potential need for multiple procedures is assessed, as patient positioning may require adjustment across different planes. This approach allows for multidisciplinary teams to be organized in advance for the intervention. As a result, operative duration, intraoperative blood loss, and the need for on-the-spot surgical decisions can be minimized. Furthermore, by simulating the surgical trajectory on a 3D model, the risk of neurovascular injury is substantially reduced from the outset. The printed model was used in multidisciplinary sessions involving orthopedic and vascular surgeons.

In the preoperative preparation on the day of surgery, general anesthesia was induced with standard monitoring. Prophylactic intravenous antibiotics (cefuroxime, 1 g) were administered pre-incision, with a plan for continued coverage for 48 h. The patient was positioned supine with a small ipsilateral pelvic bolster to optimize exposure.

A proximally and distally extended right iliofemoral (pararectal) incision was made, incorporating access to both retroperitoneal and inguinal regions in the surgical approach.

In the proximal dissection the retroperitoneal space was entered, and the right common, internal, and external iliac arteries were identified and looped with vessel tapes for protection. This proximal vascular control was planned based on the 3D model, which demonstrated proximity of the ossification to the iliac vessels.

**Figure 6:**
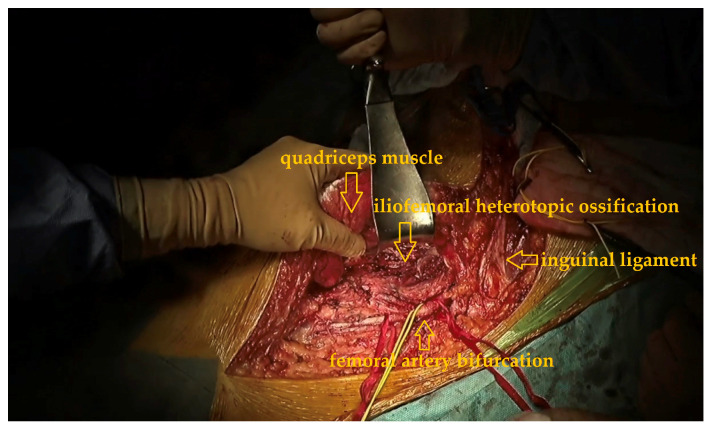
Intraoperative view of the dissected external iliac, deep femoral and femoral artery with vessel loops, inguinal ligament and the subjacent ossification.

In the distal dissection, the dissection proceeded distally into the Scarpa triangle, identifying the femoral artery and its bifurcation. The deep femoral artery was encased in dense scar tissue and was sacrificed to facilitate safe excision. The femoral vein and nerve were mobilized and protected throughout the procedure (Figure 6).

To expose and remove the ossification, the iliac attachment of the ossification was first exposed and separated from the inner iliac surface. The distal femoral attachment was then dissected. En bloc resection proved impossible, as anticipated from the preoperative simulation, due to the bulk and irregularity of the ossification.

**Figure 7:**
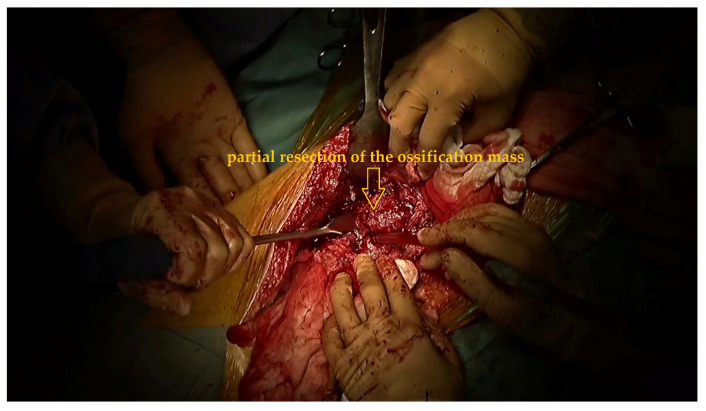
Step-by-step resection of the ossification mass in front of the right hip joint.

The bone bridge was instead sectioned into manageable fragments using a chisel and osteotome, with constant irrigation to minimize thermal injury. Sequential removal of these fragments allowed for progressive mobilization of the surrounding soft tissues. Bleeding from the cancellous bone and adjacent tissues was controlled with bone wax and bipolar coagulation (Figure 7).

Regarding the status of the hip joint, once the final fragment was removed, the hip joint capsule was inspected and found to be intact. Passive range of motion was tested intraoperatively, confirming 105° flexion, full extension, and restoration of rotational movement.

In closure two drains were placed: one in the retroperitoneal space and one in the femoral wound. Layered closure was performed, and the skin was closed with interrupted nylon sutures.

### 3.2. Stage 6—Postoperative Imaging Validation

**Figure 8:**
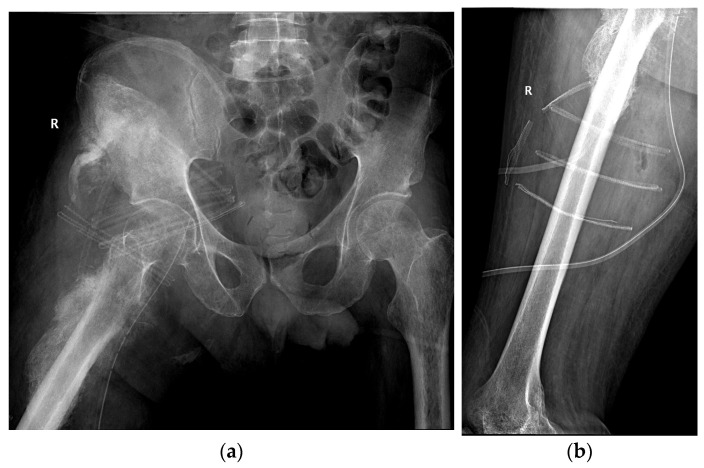
Postoperative radiographs: (**a**) Lowenstein view of the proximal femur; (**b**) lateral view of the femur (R—right side).

Postoperative radiographs confirmed complete removal of the ossification with preservation of the hip joint space and no residual bone fragments. The femoral neurovascular bundle was intact and in its expected anatomical position (Figure 8).

### 3.3. Postoperative Course

Early recovery occurred, with drains removed at 48 h. The patient remained hemodynamically stable and afebrile. As a complication, a small seroma developed at the distal wound end one week post-discharge, which was resolved by digital compression without surgical intervention. Rehabilitation consisted of early passive hip mobilization that was initiated on postoperative day 2, focusing on maintaining the intraoperatively achieved range of motion. Physical therapy was continued after discharge, with improved wheelchair transfer ability and hygiene care compared to the preoperative state. Regarding neurological status, no change in paraplegia was observed, as expected. Sensory loss remained below the T12 level.

## 4. Discussion

The workflow designed in this research primarily has clinical significance. This case demonstrates the feasibility and advantages of applying a structured, 3D-assisted workflow to the resection of massive heterotopic ossification. The results of our study are similar with those reported for the fixation of complex acetabular fractures [27]. The six-step framework validated in this study was shown to be equally applicable to a condition with a different etiology but comparable anatomical complexity.

Unlike conventional planning, which relies on 2D images and on-screen reconstructions, the life-size 3D-printed model provided tangible, tactile insights into the HO’s complex geometry and its critical relationship with the neurovascular bundle [39]. This superior anatomical understanding enabled proactive risk reduction. Furthermore, the model facilitated realistic preoperative simulation, allowing the multidisciplinary team to determine that an en bloc resection was impractical and to pre-plan a safe piecemeal strategy—transforming the surgical approach from a reactive intraoperative discovery into a proactive, rehearsal-driven process. Finally, the physical model served as a shared platform that enhanced multidisciplinary coordination, enabling orthopedic and vascular surgeons to jointly plan vessel control and dissection strategies with a confidence level unattainable with standard imaging alone.

The clinical framework we have developed requires multidisciplinary collaboration [40]. The case underscores the importance of an interdisciplinary approach for large HO resections near major vessels. The workflow’s simulation stage provided a shared platform for orthopedic and vascular surgeons to jointly plan exposure, vessel control, and dissection strategies, thereby reducing intraoperative surprises.

Our clinical intervention method has the advantage of reducing risks. Massive HO resections carry risks of neurovascular injury, excessive bleeding, and incomplete removal [41]. By anticipating these challenges via model-based simulations, we minimized these risks [42]. Intraoperative blood loss was controlled, and no major neurovascular injury occurred. The ability to foresee the need for deep femoral artery sacrifice exemplifies how preoperative 3D models can transform a surgical strategy into a proactive rather than reactive process.

In the literature review, we found that neurogenic HO surgery in SCI patients remains uncommon, with most reports describing smaller lesions or resections in less anatomically complex regions [43]. Reports of using 3D printing for HO are scarce, with most applications in tumor or fracture surgery [44,45]. Our case thus adds to emerging evidence that 3D-assisted workflows can be applied to non-fracture orthopedic pathology with equal benefits.

According to a bibliometric analysis conducted by Li et al. [46], the use of additive manufacturing in pelvic surgery is expanding, and our study aligns with this current trend. Beyond summarizing existing knowledge, our work provides a structured overview of how 3D printing can be integrated into preoperative planning and surgical management of giant iliofemoral heterotopic ossification. Preoperative assessment of such cases is notoriously challenging due to the complex pelvic anatomy.

Life-sized physical models accurately reproduce anatomical structures, thereby facilitating precise surgical simulations. In our experience, the 3D-printed anatomical model proved valuable for preoperative preparation by enabling the evaluation of surgical feasibility and guiding decisions regarding proximal and distal dissections, exposure, and resection of the ossification. Prior rehearsal of the procedure contributed to a reduction in both operative and anesthetic time. Notably, 3D printing allows for rapid production of anatomical models, often within 24 h. Our findings are consistent with the literature, which suggests that 3D modeling enhances outcomes in complex surgeries, while also eliminating the risks associated with repeated interventions [47].

The literature further demonstrates that 3D-printed models, when combined with conventional imaging, provide a superior understanding of intricate anatomy compared with 2D or digital 3D imaging alone [48]. Although high-quality 3D imaging improves surgical evaluations of giant ossifications, the reliance on 2D screen visualizations limits its full potential. In complex cases, multiple images from different planes are necessary for adequate planning, but their utility can be reduced by factors such as patient positioning, bowel gas, abdominal compression, contrast artifacts, and inconsistencies in communication between surgeons and radiology staff [49]. Our observations agree with Lim et al. [50], who reported that 3D physical models enhance the accuracy of identifying giant ossifications.

Three-dimensional (3D) printed models also serve as an effective educational resource for orthopedic residents, providing hands-on exposure to patient-specific anatomy. By replicating the exact bony structures, these models enable a clearer visualization and easier evaluation of pelvic deformities. For the surgical team, the ability to examine a life-sized, tangible 3D reproduction—one that can be rotated and inspected from multiple angles and physically handled—facilitates a more comprehensive preoperative assessment. This is particularly beneficial in complex anatomical cases, where accurate classification and surgical planning directly contribute to improved precision during the procedure.

As a single case, this study has inherent limitations. First, the findings lack standardized outcome measures that would allow for a direct comparison with procedures planned without 3D models—this reflects the nature of the report format rather than a methodological deficiency. Second, while the workflow proved feasible in this specific patient, its generalizability to other HO cases or clinical settings cannot be inferred from a single observation. Third, although the model required approximately 20 h for processing and printing, this delay is acceptable in the elective context of a mature HO resection. The cost of materials was relatively low (PLA filament), but broader implementations would require assessments of institutional resources and expertise. These observations are specific to this case and warrant confirmation in future studies with larger patient cohorts and validated outcome measures. In this single case, the use of the 3D-printed model facilitated preoperative preparation. However, in this situation, we lacked standardized outcome measures to objectively compare results with procedures planned without 3D models. The clinical benefits observed here should therefore be considered anecdotal and specific to this patient, warranting confirmation in larger comparative studies. This represents an inherent limitation of the case report format, not a methodological deficiency of the proposed workflow. Future larger-scale studies incorporating validated functional and surgical outcome measures would be valuable to quantitatively establish the benefits observed here. While technology continues to advance, becoming increasingly affordable and accessible, significant challenges remain regarding regulation, safety, and broader clinical integration.

Future research directions include potential improvements. One research direction consists of the intraoperative incorporation of augmented reality (AR) overlays based on the virtual model. Another research direction is the use of biocompatible printing materials to create intraoperative templates for guiding osteotomy. The application of the workflow to bilateral or multiple HO resections in patients with SCI can be studied. Another research direction consists of developing standardized indicators that would allow for a direct comparison between surgeries planned with the aid of 3D models and those using conventional methods. Such measures could also be incorporated into frameworks assessing healthcare practices and sustainability [51]. Furthermore, advancing research into safety, security, and regulatory standards will be essential to support the widespread adoption of 3D printing in orthopedic surgery.

## 5. Conclusions

The development of a six-stage, 3D-assisted clinical workflow of the surgical management of giant iliofemoral heterotopic ossification provided tangible benefits in anatomical understanding, surgical safety, and multidisciplinary planning. Key advantages included: precise mapping of HO relative to neurovascular structures, realistic preoperative rehearsal, informing the surgical sequence, safe resection with restoration of hip mobility and improved patient care.

Given the complexity of such cases, this workflow offers a valuable model for integrating advanced imaging and additive manufacturing into orthopedic surgical practice beyond fracture care.

## Data Availability

Data is contained within the article.
